# Redox Active Molecules in Cancer Treatments

**DOI:** 10.3390/molecules28031485

**Published:** 2023-02-03

**Authors:** Višnja Stepanić, Marta Kučerová-Chlupáčová

**Affiliations:** 1 Laboratory for Machine Learning and Knowledge Representation, Ruđer Bošković Institute, Bijenička 54, 10000 Zagreb, Croatia; 2 Department of Pharmaceutical Chemistry and Pharmaceutical Analysis, Faculty of Pharmacy in Hradec Králové, Charles University, Ak. Heyrovského 1203/8, 500 05 Hradec Králové, Czech Republic

Cancer is one of the leading causes of death worldwide, with nearly 10 million deaths in 2020 [1]. Redox active molecules in the diet, dietary supplements, or in approved drug preparations are used to prevent and treat cancer.

The main objective of this Special Issue, “Redox Active Molecules in Cancer Treatments”, in the journal *Molecules* is to present the results of in vitro, in vivo, and/or in silico studies on the biological effects and activities of anti- and pro-oxidant molecules observed in original research studies or collected and discussed in review articles. This goal is achieved by compiling seventeen articles. They present antioxidative or targeted oxidative effects of miscellaneous small-molecular-weight compounds or proteins against a variety of cancer types:

- An endogenous compound—melatonin [2].
- Natural plant compounds (naringenin [3], papaverine [4], polyphenols isolated from *Myrciaria trunciflora* [5] or *Anneslea fragrans* [6], and seed-derived peptides [7]), natural compounds also found in animals (melatonin [2,8]), and peptides as well as proteins from Jellyfish venom [9].
- Synthetic compounds, i.e., alkyl thiols [10], dimethyl sulfoxide [11], metformin and S63845 [12], the ruthenium complex [Ru(Phen)_3_]^2+^ [13], and copper-based compounds—Casiopeinas [14].
- Different formulations, i.e., peptide fractions from germinated soybeans conjugated to Fe_3_O_4_ nanoparticles [15] and astaxanthin microparticles in combination with pentoxifylline [16].
- Proteins (aquaporins [17]) and nuclear factor erythroid-2-related factor 2 (NRF2) [8].

The studies explored diverse anticancer mechanisms of action of redox-active molecules in association with specific signaling pathways by using in vitro and in vivo methods. Some studies investigated the use of redox-active compounds to alleviate radiation-induced fibrosis, which is a side-effect of radiotherapy [16], or to detect oxygen in vitro and in vivo [13]. Most studies examined the effect of the tested compounds on cancer cell viability/proliferation assays [2,3,4,6,11,12] and/or analyses of reactive oxygen species concentrations [2,3,6,11,15,16]. Some other studies used in vitro assays such as cell cycle analyses [2,3,4,9], DNA fragmentation assays [3,9], analyses of the expression of apoptosis-related proteins and/or genes [9,11,12], etc. The two included studies are based on the application of state-of-the-art chemoinformatic analysis and modeling approaches—molecular docking and molecular dynamics [7,18].

The whole series of thirteen experimental investigations and one computational study is accompanied by three review articles focusing on aquaporins as redox regulators in breast cancer [17], natural compounds affecting ferroptosis [18], and modulation of NRF2 expression at the mRNA and protein levels [8].

We hope that readers will enjoy the book and glean interesting and useful information from the particular studies.
